# Diarrhoeal disease in children due to contaminated food

**DOI:** 10.2471/BLT.16.173229

**Published:** 2016-11-25

**Authors:** Martyn D Kirk, Frederick J Angulo, Arie H Havelaar, Robert E Black

**Affiliations:** a National Centre for Epidemiology and Population Health, The Australian National University, Canberra, Australian Capital Territory 0200, Australia.; b Division of Global Health Protection, Centers for Disease Control and Prevention, Atlanta, United States of America (USA).; c Emerging Pathogens Institute, University of Florida, Gainesville, USA.; d Institute for International Programs, Johns Hopkins Bloomberg School of Public Health, Baltimore, USA.

In December 2015, the World Health Organization (WHO) released estimates of the burden of human disease attributable to consumption of food contaminated with 31 infectious agents or chemicals.^1^ The report concluded that exposure to contaminated food worldwide in 2010 resulted in 600 million episodes of illness (95% uncertainty interval, UI: 420–960 million), 420 000 deaths (95% UI: 310 000–600 000) and 33 million disability-adjusted life years (DALYs) (95% UI: 25–46 million).^1^ The numbers were based on 4.6 billion cases of diarrhoea (95% UI: 3.5–6.5 billion) and 1.6 million deaths due to diarrhoea (95% UI: 1.3–1.9 million) that occurred worldwide in 2010, similar to numbers occurring in later years.^2^

A key element of the estimation was attributing a proportion of the diarrhoea deaths to foodborne transmission of infections. A structured expert judgement was used to apportion transmission modes for individual pathogens, by estimating that 29% (95% UI: 22–36%) of 11 key bacterial, viral and protozoal causes of diarrhoea were foodborne.^3^ Food contaminated with these 11 agents resulted in 548 million episodes of diarrhoea (95% UI: 370–888 million) and 200 000 deaths (95% UI: 137 000–287 000) in 2010. Of these, 217 million infections (39%; 95% UI: 29–38%) were in children younger than 5 years of age.^4^ This disproportionate burden of foodborne diarrhoeal disease in young children is evident in the high rate ratio of DALYs in children younger than 5 years compared with older children and adults (ratio: 11.6; 95% UI: 8.4–15.6). Among children younger than 5 years, foodborne transmission of the 11 agents could have constituted as much as 16% of the estimated 578 000 deaths due to diarrhoea (95% UI: 448 000–750 000), updated to 2013.^5^

Regionally, Africa had the greatest burden of diarrhoeal disease from contaminated food among all age groups: 687 DALYs (95% UI: 369–1106) per 100 000 population compared with 229 (95% UI: 160–323) per 100 000 globally and 23 (95% UI: 13–33) per 100 000 in North America.^4^ These differences suggest that implementation of preventive measures in low- and middle-income countries could prevent substantial foodborne disease. Furthermore, the WHO disease burden estimates were conservative, because for methodological reasons they largely excluded diarrhoeal disease associated with human immunodeficiency virus (HIV) infection. It is likely that HIV-infected persons experience a substantial burden of infection from contaminated food, making food safety important for this vulnerable group. In addition, these estimates have not yet captured the effects of foodborne disease or subclinical enteric infections on malnutrition and its subsequent health and development outcomes.^6^

Globally, diarrhoeal infections with *Salmonella* species (including invasive infections), enteropathogenic and enterotoxigenic *Escherichia coli*, norovirus and *Campylobacter* species were responsible for the greatest burden of foodborne disease. Food safety measures that would be effective against these enteric pathogens are likely to be similar, at least at the food preparation stage. Credible evidence to guide interventions is scarce, however. Contamination of food can occur anywhere along the chain of production and preparation, from where a food or ingredient is grown, harvested, processed, transported and sold, through to where it is prepared before consumption. Hygienic preparation and storage of food in the home is particularly important for young children, but so too are systemic improvements in food supplies, such as pasteurization of milk.^7^ Food safety education for consumers has been shown to affect behaviour change, but there are many other factors in preventing disease, including interventions aimed at the food processing, service and retail sectors.^8^

Before the 2015 WHO report,^1^ the disease burden due to contaminated food, and therefore the importance of food safety, was somewhat neglected. Globally, much attention has been given to improving water and sanitation; rightly so, as this is a vital factor in attempts to decrease the rates of diarrhoeal disease due to all causes. Contaminated water plays a role too in foodborne disease when it is used in the preparation of food. However, the lack of specific attention on the importance of foodborne disease has meant that governments, industry, donors and funding agencies have not prioritized improving food safety. International agencies, such as WHO and the Food and Agriculture Organization, along with many other international agencies and nongovernmental organizations, have a long history of working to improve food safety. To have maximal effect, these efforts need to be targeted at contaminated foods and at populations at higher risk of disease. To date, the majority of food safety efforts have been too general and non-specific to succeed in interrupting possible disease transmission pathways. It is likely that there is a strong correlation between food insecurity in a geographic area and the risk of foodborne disease (for example, if families have to eat foods that are unsafe), although this is poorly quantified.^9^

The WHO estimates of the foodborne disease burden highlight the need for attention on improved food safety that will specifically prevent infections in children.^10^ A key strategy to prevent foodborne infections in children younger than 6–23 months of age, who have very high rates of diarrhoeal illness and death, is to improve the safety of complementary foods introduced to infants to supplement breast or formula feeding. Contamination of these foods may result in serious enteric infections, despite the partial protective effects of breastfeeding.^7^^,^^10^^,^^11^ Food-producing animals often live in close proximity to humans; additional efforts are required to prevent exposure of young children to the excreta of these animals, which may cause zoonotic infections. Efforts to reduce the disease burden and improve the nutrition of young children may not be effective without concomitant controls on exposure to foodborne, waterborne and zoonotic pathogens.

If we are to reduce the high burden of foodborne illness in young children, we need high-level advocacy for research that will identify and validate specific food safety interventions. A research agenda to address the lack of evidence could start with better knowledge of the disease burden: identifying the localized burden of foodborne disease in low- and middle-income settings within specific countries; development of disease burden estimates for specific foods that may present high risks; and elucidation of the human disease burden from chemicals in food. Despite the high burden of diarrhoeal diseases in children, there appear to be no intervention studies identifying how much these diseases could be prevented by safer food. This contrasts with the many studies assessing the effects of improved water and sanitation on diarrhoeal disease.^12^ For better disease control we need to assemble a credible information base on the effects of food safety interventions to prevent foodborne diarrhoea in children. This would inform interventions targeted at mothers and children, along with the development and implementation of effective surveillance systems to measure progress in reducing diarrhoeal diseases. An economic analysis of the costs of foodborne disease and the benefits of interventions would support these efforts. Finally, research is needed to develop vaccines for use in animals and humans against agents that are commonly foodborne, such as norovirus.
